# A Regional Scoping Review of School‐Based Nutrition Interventions Conducted Across Nordic Countries

**DOI:** 10.1002/hsr2.72279

**Published:** 2026-04-05

**Authors:** Basil H. Aboul‐Enein, Stephen Gambescia, Nada Benajiba, Norah Eid Aljohani, Teresa Keller, Patricia J. Kelly

**Affiliations:** ^1^ College of Arts & Sciences, Health & Society Program University of Massachusetts Dartmouth North Dartmouth Massachusetts USA; ^2^ London School of Hygiene & Tropical Medicine, Faculty of Public Health and Policy London UK; ^3^ Health Administration Drexel University Philadelphia Pennsylvania USA; ^4^ Ibn Tofail University–CNESTEN, Joint Research Unit in Nutrition and Food, RDC‐Nutrition AFRA/IAEA Rabat Kenitra Morocco; ^5^ Department of Clinical Nutrition, College of Applied Medical Sciences Taibah University Medina Saudi Arabia; ^6^ School of Nursing New Mexico State University Las Cruces New Mexico USA; ^7^ College of Nursing Thomas Jefferson University Philadelphia Pennsylvania USA

**Keywords:** Nordic countries, nutrition, Scandinavia, school

## Abstract

**Background and Aims:**

While Nordic countries have a relatively high socioeconomic status population, they are challenged by the health deficit of rising obesity and overweight among young people. The aim of this regional scoping review is to identify best practices for designing and implementing effective school nutrition programs in Nordic countries and identify gaps for further research.

**Methods:**

Using the PRISMA‐ScR guidelines, a scoping review was conducted across nine academic databases between 1980 and May 2025.

**Results:**

Fifty‐three studies met the inclusion criteria, from small pilot interventions to large national or international cohorts involving thousands of students across the five Nordic countries.

**Conclusion:**

Most intervention studies were multicomponent in nature, combining individual student‐focused education, changes to the school environment, and engagement with families or the wider community. The reviewed interventions consistently demonstrated positive impacts on nutrition knowledge, dietary behaviors, and in some cases, physical health indicators, though effect sizes and sustainability varied. Some schools have the advantage of a national nutrition education framework that gives consistency to the nature and extent of nutrition promotion, but all schools have found ways to include nutrition education programs in the curriculum. Government or private entity provisions of healthy meals or snacks have a significant effect on the improvement of youth nutrition, especially for students with limited familial resources.

## 1 Introduction

The growing public health issues surrounding childhood obesity have led to a heightened emphasis on schools as a vital avenue for fostering healthier dietary habits among students and their families [1]. Children typically spend a significant amount of time in educational settings and often consume at least one meal at school during the day. Such meals and snacks could be from one or a combination of food brought from home, government‐supported foods served, or food offered by the schools from private vendors. The importance of school in a child's life makes schools an ideal target for initiatives meant to teach healthy eating practices. Students are influenced by teachers and other adults at school, as well as their age cohorts and older students, in a range of behavioral activities. Nordic countries, facing rising rates of childhood obesity, have pioneered innovative approaches to school nutrition targeting a range of student needs and populations. These nations have established programs to guarantee every child access to a healthy, well‐balanced meal, regardless of their economic background. This illustrates how effective macro‐level policies can tackle these complex issues directly. By focusing on local, sustainable food sources and adhering to rigorous nutritional guidelines, schools in Nordic regions provide nourishment, cultivate healthier future generations, and set a global standard for school nutrition initiatives [2].

School meal programs are widely recognized as valued community activities, serving 61% of primary school students in affluent countries. While not given, food benefits understandably are less available in schools in lower‐income nations. Many countries are grappling with rising rates of childhood obesity alongside ongoing challenges of malnutrition and food insecurity, particularly in regions where access to food is hindered by geographic or economic constraints. Globally, school nutrition programs face significant obstacles. In certain areas, children arrive at school hungry, which adversely affects their concentration and ability to learn, while in others, the prevalence of highly processed, calorie‐rich meals and snacks fuels the childhood obesity epidemic, compromising students' academic and physical development. Striking a balance between addressing hunger and promoting long‐term health is a multifaceted challenge that remains unresolved in many contexts [3].

The nature and extent of school nutrition programs range from educating teachers about the science behind good nutrition, teaching students about the benefits of healthy food consumption, educating parents about serving and preparing healthy foods for their families, motivating school administrators to make healthy food choices from vendors, and advocating government to provide healthy food in school breakfast or lunch programs. Schools face numerous challenges in creating optimal and agreeable nutrition programs accessible to all children. Many school‐based nutrition initiatives struggle with implementation, keeping to the integrity of an evidence‐based program, sustainability, and demonstrating measurable improvements in students' health outcomes [4].

Studies have shown that school nutrition interventions can positively impact dietary choices, but their effectiveness often varies based on factors like intervention design and delivery, as well as socioeconomic and environmental influences [5]. Single interventions, such as offering more fresh fruit on the school menu, are less effective than programs that feature a comprehensive approach to improving school nutrition using multiple intervention strategies, such as nutritional education, menu changes, and parental involvement. School nutrition programs are also subject to limitations related to local resources, changing levels of government support, where to place this in the curriculum and its time constraints, or diminishing interest among schoolchildren and their families [6]. Other threats to program success include the need for close collaboration among school personnel and between the school and the community [7]. Other threats include consistency and sustainability as local resources, and parental involvement may vary among program sites, and the increasing exposure that children have to marketers of unhealthy food products—at increasingly younger ages.

Still, some countries are experiencing success with a thoughtful, culturally based approach to school nutrition programs. Nordic countries have embarked on an ambitious initiative to improve school nutrition based on regional availability, palatability, and cultural acceptability. This is evidenced by a collaborative endeavor in 2009 via *OPUS* (Optimal Well‐being, Development, and Health for Danish Children through a Healthy New Nordic Diet) [2]. This program aims to provide environmentally friendly school foods, foods that are palatable to schoolchildren, and locally sourced. The diet focuses on traditional Nordic food such as fish, shellfish, regional fruits and vegetables, and free‐range livestock supplemented with wild game [8].

This scoping review examines the peer‐reviewed literature on the nature and extent and effectiveness of various school nutrition interventions used in the Nordic countries of Denmark, Sweden, Norway, Iceland, and Finland from 1980 to May 2025. This starting year coincides with the Nordic Nutrition Recommendations (NNR), first published in 1980, and updated about 4–8 years, that provides the scientific basis and subsequent teaching and information awareness for these national recommendations [9]. Furthermore, it gives a half a year short of a 45 years search on peer‐reviewed literature on this topic. Established and emerging programs and practices are reviewed to identify successful strategies that might be adapted elsewhere.

### 1.1 Aim

The aim of this scoping review bridges an existing knowledge gap in the published peer‐reviewed literature and characterizes and evaluates the available scientific evidence pertaining to school‐based nutrition interventions conducted across Nordic countries exclusively or in some parts thereof from a multinational study. This review aims to find the best practices for designing and implementing effective school nutrition programs demonstrated by school nutrition interventions and outcomes in Nordic countries.

## 2 Methods

In line with the objective of examining school‐based nutrition interventions conducted across Nordic countries, we applied scoping review strategies for the search, selection, and reporting of studies [10]. Given that methodological quality assessment is not a prerequisite for scoping reviews, we did not appraise the included studies [11]. For the purposes of this review, Nordic countries included: Denmark, Finland, Iceland, Norway, and Sweden.

### 2.1 Selection Criteria

**Figure 1:**
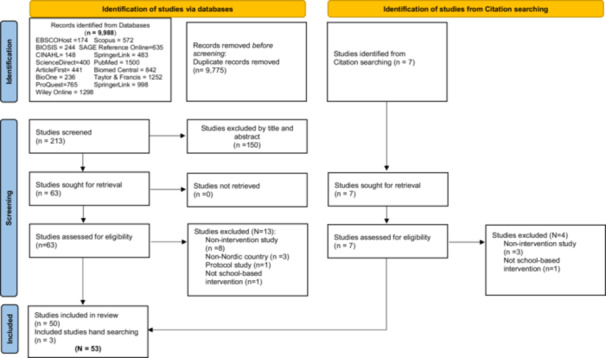
Search flow diagram following PRISMA 2020 guidelines.

The Population, Intervention, Comparison, Outcomes and Study (PICOS) design guidelines were incorporated to develop the research question: “Do children, adolescents, parents, or school teachers/staff members in Nordic countries (P) that are offered school‐based nutrition interventions (I) have improved health and wellness parameters (O) compared with those that do not participate in school‐based nutrition interventions(C)?” and subsequent inclusion and exclusion criteria (see Table 1). Peer‐reviewed articles published in English were included. Articles were included if they involved the five Nordic countries' school student populations, in part. Interventions reported outside traditional peer‐reviewed articles were excluded in this review. Qualitative intervention studies and those assessing program implementation were included if they also included outcomes. Process development studies were excluded. The search was conducted in the Spring of 2025 and the results communicate literature published between 1980 and May 2025. Figure 1 provides the PRISMA flowchart leading to selected studies for this review.

**Table 1:** PICOS criteria for inclusion and exclusion of studies.

Parameter	Inclusion criteria	Exclusion criteria
Population	School‐aged students (i.e., around 6 years old and above), teachers, staff, or parents who were examined in any Nordic country within the school setting Multinational studies on school‐based nutrition interventions that involved Nordic countries in some way	Students who are not of school‐age Students who are not studying in a Nordic country Students undergoing medical nutrition therapy‐based diets or sports‐focused diet
Intervention type	Any kind of school‐based intervention that addresses nutrition‐related aspects, including:	Interventions that are not based on school facilities Interventions that do not address nutrition‐related outcomes
	Educational interventions Environmental Interventions Multicomponential Interventions	
Comparators	Pre‐intervention, baseline nutrition‐related variables (i.e., anthropometric measures, biochemical parameters, nutrition‐related knowledge, dietary habits, perceived hunger) of student groups who were:	N/A
	Control: received no intervention Received partial intervention, e.g., educational intervention only vs. multicomponential intervention	
Outcomes of Interest	Changes in anthropometric outcomes, e.g., BMI for age, height for age Changes in biochemical outcomes Changes in nutrition‐related knowledge Changes in meeting the dietary macronutrient and/or micronutrient recommendations Changes in adherence to healthy dietary habits and avoidance of unhealthy ones changes in risks of nutrition‐related diseases, e.g., obesity or iron‐deficiency anemia changes in short‐term hunger	Non‐nutrition related outcomes
Language	English	All other languages
Study Type	Experimental intervention studies with measured outcomes Peer‐reviewed original research articles Original research conference publications	Non peer‐reviewed articles Non‐numeric/categorical assessments or qualitative studies Commentaires Narratives Protocols Communications Nonintervention‐based studies White papers Similar article types
		Gray literature

Abbreviations: BMI, body mass index; N/A, not applicable.

### 2.2 Search Procedures

For this scoping review, we followed PRISMA Extension for Scoping Reviews [12] and began with a comprehensive search within biomedical bibliographic databases using a combination strategies of medical subject heading keywords, terms, phrases, and Boolean operators (see Supporting Information S1). The following 14 databases were searched: EBSCOHost, BIOSIS, CINAHL, ScienceDirect, ArticleFirst, Biomed Central, BioOne, ProQuest, SAGE Reference Online, Scopus, SpringerLink, PubMed, Taylor & Francis, and Wiley Online. Search strategies were adapted according to the indexing systems of each respective database (see Supporting Information S1).

## 3 Results

The included studies (*N* = 53) represent a diverse range of school‐based nutrition and health promotion programs conducted across Nordic countries exclusively or in some parts thereof in a multinational study (see Table 2). The peer‐reviewed publications span from 1982 to 2024, reflecting over four decades of research in this area. Notable peaks in publication occurred in 2012 and 2021 [13, 14, 15, 16, 17, 18, 19, 20, 21, 22], each with five studies, while significant gaps were observed between 1993 and 2003, as well as in 2004, 2007, and 2013, with no studies from this list published during those years. A total of 16 studies were conducted in Denmark, 15 in Norway, 7 in Finland, and 7 in Sweden. Only three studies were conducted in Iceland. The remaining five studies were multinational, involving one or more of these Nordic countries.

**Table 2:** Intervention characteristics of included studies (*N* = 53).

Author, year/country	Goal	Design	Participants	Intervention content	Theoretical framework	Results	Discussion
Abdollahi et al., 2021/Finland	Assess effect of educational escape game vs. self‐study method as a nutrition knowledge intervention	RCT over 1 h period	130 students (11–14 years old) from 3 schools (1 Finland, 2 UK); IG: 68; CG: 62	IG: participants played nutrition education escape game with focus on plant‐based protein sources; CG: participants received educational leaflet with identical content	N/R	Both IG (*p* < 0.001) and CG (*p* = 0.002) had improved overall knowledge scores	Educational escape game may enhance motivation for learning, possible behavior change; use warrants further investigation
Andersen et al., 2014/Denmark	Investigate effects of introducing school meals based on national policy, *OPUS (Optimal well‐being, development and health for Danish children through a healthy New Nordic Diet*), of covering lunch and all snacks during school day	Cluster‐RCT, cross‐over design over two 3‐month periods	834 children aged 8–11 years old from 46 school classes at 9 schools	During 3‐month NND intervention period, children offered mid‐morning snack, and libitum hot lunch meal, afternoon snack served in a small bag	N/R	During NND period children had higher intakes of cheese, potatoes, fish, eggs, vegetables, beverages, lower intakes of bread, fats than in control period (all *p* < 0.05); no difference in mean energy intake; differences in vitamin D/iodine from higher fish intake	Children's overall dietary intake improved when usual lunches replaced by school meals following the NND principles
Anttonen et al., 2011/Finland	Study effect of a dietary intervention on schoolchildren's eating habits and LF values of teeth	3‐arm, 1‐year RCT	769 7th grade students from 12 schools in 3 cities	IG 1: School lunch intervention; IG 2: school lunch plus oral hygiene intervention (education, training to remove plaque, counseling); CG: no intervention	N/R	Frequency of eating a warm meal, drinking water at school to quench thirst increased in IG but decreased in CG schools (*p* < 0.001 and *p* = 0.005); LF molar values decreased in schools with dietary intervention only (*p* = 0.024)	Intervention long enough to show improvement in eating/quenching thirst habits; some decrease in LF values of molars
Ask et al., 2006/Norway	Evaluate if breakfast served in secondary school can improve dietary habits and school performance	2‐group pre–post‐intervention × 4 months	54 lower secondary school students	IG: students offered a free breakfast at start of school day; CG: no free breakfast; both groups educated re: importance of healthy eating, ability to evaluate dietary intake	N/R	1 week after start, IG returned to normal breakfast pattern; CG had increase in lunch intake (*p* < 0.01); IG males had improved food pattern (*p* < 0.01). Body mass index significant increase in CG males and females (*p* < 0.01 males, *p* < 0.05 males), but not IG; IG males had improved school contentment (*p* < 0.05).	Students served breakfast for 4 months had dietary intake changed to more healthy profile; weight gain reduced
Beinert et al., 2021/Norway	Present learning activities of curriculum, *LifeLab Food and Health*, and how 6th and 9th grade students experienced them	Mixed methods	Faculty and 43 students	Focus groups with teachers and students conducted at three local schools to identify learning tasks for FH; results used to develop 6 learning tasks which were tested at 2 local schools	N/R	Six learning tasks developed: rank pictures of food items from least to most nutritious; identify/label two different loaves of bread; identify sugar content of different items; concretize “five a day” recommendations for F&V; read text on food and sustainability, nutrition, health, or food choices; quiz on previous topics	Specific learning tasks complement current teaching practices and may be considered as “best practice” in FH education; provide links to public health nutrition in future Home Economics education
Bere et al., 2006a/Norway	Report effect of school‐based F/V intervention, *Fruits and Vegetables Make the Mark*	Random assignment of schools to IG and CG – 1 year effect	369 6th–7th grade students from 19 schools; IG: 190; CG: 179	3 parts: home economics class component, parental involvement, school fruit subscription	Social Cognitive Theory	At Follow‐up 1 and 2, strong intervention effects for F/V intake (effect sizes were 0.6 and 0.5); sustained effect can partly be explained by greater participation rates in the School Fruit Program (standard paid subscription)	Providing pupils F/V at school at no cost for parents is an effective strategy to increase schoolchildren's intake of F/V
Bere et al., 2006b/Norway	Report effect of school‐based F/V intervention, *Fruits and Vegetables Make the Mark*	Random assignment of schools to IG and CG × 4 months	369 6th–7th grade students from 19 schools; IG: 190; CG: 179	3 parts: home economics class component, parental involvement, school fruit subscription	Social Cognitive Theory	No effect for F/V eaten at school or all day, neither at Follow‐up 1 or 2; significant differences between IG and CG in awareness of five‐a‐day recommendations only	Intervention rated as very good by teachers and pupils; however, did not succeed in changing preferences for or accessibility of F/V
Bere et al., 2005/Norway	Report effect of *Norwegian School Fruit Program*, providing schoolchildren with free fruit or vegetables every school day and the effect of an existing fee‐based fruit program	3‐group cluster randomized trial × 9 months	795 11th–12th grade students	3 program groups: free fruit, at school, paid subscription fruit program, no fruit program	N/R	Students at free fruit schools had significantly higher intake of fruit and vegetables at school than those at paid fruit and no fruit schools (*p* < 0.001)	Existing School Fruit Program appears to increase intake among the subscribers, but also to increase existing difference in consumption patterns among subscribers and nonsubscribers
Bere et al., 2014/Norway	Assess if increased consumption of fruits and vegetables from free school fruit has an impact on future weight status.	Cluster randomized trial, 3‐ and 7‐year f/up	1950 10–12‐year‐olds from 31 schools (IG: 2 schools; CG: 29 schools)	Participation in the Fruits and Vegetables Make the Marks (FVMM) Project. Received free school fruit with no parental payment for 9 months	N/R	At f/up #1, no significant difference between IG and CG in weight, body mass index, or perceived weight status; at f/up #2 difference in overweight (*p* = 0.04); after adjusting for school, sex, grade level, parent education, association not significant	Results suggest free fruit at school might help prevent future excessive weight gain; results are limited by low participation rate
Bergh et al., 2012/Norway	Evaluate mid‐ and final‐study effect of a multicomponent intervention, *HEalth In Adolescents (HEIA)*, on theoretically informed determinants of physical activity/dietary/sedentary behavior	School‐based RCT × 20 months	1418 11‐year‐old students	A mix of individual‐, group‐, and environmental strategies and components involving dietary and physical activity	N/R	At mid‐way enjoyment (*p* = 0.03), perceived teacher social support (*p* = 0.003), self‐efficacy (*p* = 0.05) were higher in IG; weight status moderated effect on self‐efficacy, with a positive effect observed among the normal weight only; at post‐intervention results sustained for teacher social support (*p* = 0.001), negative effect was found for self‐efficacy (*p* = 0.02)	Intervention affected both psychological and social‐environmental determinants; teachers' social support potential mediator of change; overweight adolescents might be in need of specially targeted interventions
Bestle et al., 2024/Denmark	Evaluate the effectiveness of the intervention “Are You Too Sweet?” in reducing the intake of discretionary foods and drinks among children starting school	3.5‐month two‐arm cluster RCT	153 children aged 5–7 years	Three‐part intervention involving an extended consult with the school health nurse, from 30 to 35 min, that is, 5 min focusing on discretionary foods and drinks; A use‐at‐home box containing intervention materials; and invitation to join a private Facebook group to provide prompting posts and support interaction among participating parents	The Social Cognitive Theory	Significant reduction in the children's intake of total discretionary foods and drinks or discretionary foods alone was observed between the intervention and control group, while a decreased intake of discretionary drinks of 40.9% (*p* = 0.045) was observed compared with control. Secondary subgroup analysis showed that children of parents with shorter educational level significantly reduced their intake of added sugar by 2.9 E% (*p* = 0.002).	Results indicate that multicomponent interventions involving school health nurses may have some effects in reducing, especially, discretionary drinks
Bjelland et al., 2011/Norway	Evaluate effect of a multicomponent intervention, *HEalth In Adolescents (HEIA)*, on intake of sugar‐sweetened beverages (SSB) and screen time	Cluster randomized controlled pre–post study	1465 6th grade students; IG: 566; CG: 1014; 349 parents with process evaluation	Mixed individual‐, group‐, environmental activities, such as lessons with student booklet, classroom F/V breaks, equipment for recess activities, active commuting, parents' fact sheets, teacher course	N/R	Time spent on TV/DVD (week *p* = 0.001, weekend *p* = 0.03), computer/game‐use (week *p* = 0.004, weekend *p* < 0.001), intake of SSB during weekend days (*p* = 0.04), significantly lower among IG girls than CG	Initiatives appeared to change girls' behavior only; exploration of potential effects in subgroups is important; future work might include issues of gender, weight, parent involvement to enhance effectiveness
Bonde et al., 2018/Denmark	Evaluate *We Act* implementation fidelity, interacting context factors of a health‐promoting school intervention	Process evaluation	269 students in Grades 5–6, 22 teachers over 12 classes	Focus on dietary habits, physical activity, well‐being, and social capital using Investigation, Vision, Action & Change (IVAC) health educational methodology	N/R	Implementation fidelity high for Initial phases, low for Action & Change phase was low; little change occurred in schools; pupils' presentation of their visions did not work; weak parental and school components; poor fit to school‐year plan, weak management support	Intervention did not result in change; time for school prep and competence development was too low and should be considered in future evaluation
Christensen et al., 2019/Denmark	Assess effect of free breakfast club on dietary habits of students	4‐month cluster RCT	318 students from 4 vocational schools	IG schools had free whole‐grain breakfast every school day; CG schools had no free breakfast	N/R	Proportion of students who had breakfast increased significantly in IG compared to CG schools for first follow‐up (*p* = 0.0149); effect attenuated at the second follow‐up; breakfast whole grain product intake increased among students in IG compared to CG at first (*p* = 0.0079) and second follow‐up (*p* = 0.0317); no change in snacking	Provision of free breakfast at vocational schools can improve quality of breakfast; sustainability a critical issue
De Henauw et al., 2015/Sweden and seven countries	Investigate etiology of childhood obesity, develop primary prevention program, Identification and prevention of *Dietary‐ and lifestyle‐induced health EFfects In Children and infantS (IDEFICS)*, and report on effects of on body fatness	Mixed method, primary prevention trial × 2 years	16,228 children from 2 to 9.9 years old	Intervention modules addressed community, school, and parental level, focusing on diet, physical activity, stress‐related lifestyle	N/R	Prevalence of overweight and obesity increased in both CG (8.0%–22.9%) and IG (19.0%–23.6%)	This primary prevention program for childhood obesity has not been successful in reducing the prevalence of overweight obesity in the target population
Elinder et al., 2012/Sweden	Improve eating habits, physical activity, self‐esteem, and promote healthy weight in children aged 6–16 years in the *Stockholm County Implementation Program*	Quasi‐experimental design with 9 schools × 2 years	813 students from Grades 2, 4, and 7	Logic model determined strategies consisting of mixture of individual, group, and environmental‐level actions	Socioecological Model of Health	High program fidelity demonstrating feasibility, but fidelity to school action plans was 48%; significant (*p* < 0.05) impacts in school health practices/environments; at student level no significant intervention effects seen	Changes by school staff challenging to sustain over time, with little impact on student behaviors/weight; perhaps better student outcomes with more evidence‐based approach with action plans with stepwise implementation
Elinder et al., 2020/Sweden	Optimize meals for minimum deviation from current food supply, reduce greenhouse gases, ensure nutritional adequacy without increasing cost, *The OPTIMAT Intervention Study*	Pre–post design × 6 weeks with interrupted time series analysis	4 primary schools (up to Grade 9)	Students received usual menu × 3 weeks, then isocaloric menu with 181 food items × 3 weeks	Social Cognitive Theory	No significant changes in mean food consumption or plate waste found in analysis between two periods	Results demonstrate that climate‐friendly and nutritionally adequate menu plans for Swedish schools can be designed through optimization and experienced meal planning
Eriksen et al., 2003/Denmark	To measure the effect of school/vegetable subscription on children's intake	Field trial × 5 weeks	Children 6–10 years old; IG: 804 from 4 schools; CG: 689 from 3 schools	IG children receive a piece of F/V every day during mid‐morning break	N/R	Both subscribers and nonsubscribers had increased fruit intake (*p* = 0.019; *p* = 0.008) pieces/school day; no change in vegetable intake	5 weeks with subscription affected both subscribers and nonsubscribers to increase fruit intake; may indicate subscription had additional effect of stimulating parents of nonsubscribers to supply children with fruit
Fuller et al., 2017/Denmark	To modify *FIFA 11 for Health program* to the European situation, and assess effects on health knowledge, well‐being in Danish children	A 2‐cohort study	546 children (10–12 years) IG: 7 schools with *n* = 402; CG: 2 schools with *n* = 144	Football‐based program developed in 2009 to engage 10–13‐year‐old children in sub‐Saharan Africa to increase physical activity and health knowledge = 11, 90 min sessions focused on single health message linked to football skill	N/R	Health knowledge significantly increased (*p* < 0.05) greater for IG (11.9%) than CG (2.6%); significant (*p* < 0.05) between‐group differences obtained for 8 of 10 health topics (6.1%–20.2%)	Modified program demonstrated positive effects on children's health knowledge and social dimension of well‐being, providing evidence that football‐ based education program can be effective in European schools
Goswami et al., 2021/Norway	Assess how a daily combination of PA and diet, the *Health Oriented Pedagogical Project*, affect anthropometric measures	3 years	917 4th graders (IG: 614; CG: 303) from 9 schools	45 min of daily physical activity during teaching– active learning, 4–6 × week vegetable	N/R	Daily PA, 4–6 × week vegetable intake had positive influence on muscle (*p* = 0.028), bone mass (*p* = 0.015)	Daily PA can counteract effects of unhealthy diet on weight/fat mass; daily PA and regular fruit lowers weight/fat mass in overweight children
Grydeland et al., 2014/Norway	Investigate effects of multicomponent school‐based intervention, *HEalth in Adolescents*, targeting physical activity, sedentary/dietary behaviors on anthropometric outcomes	Cluster RCT × 20 months	11‐year‐old students (IG: 566 from 12 schools; CG: 1014 from 25 schools)	Multilevel approach with principals, teachers, health services, parents; activities to promote healthy diet, healthy choices, physical activity during school/leisure time, reduce screen‐time	N/R	Beneficial effects for girls BMI (*p* = 0.02), BMIz (*p* = 0.003), not in boys; positive BMI effect (*p* = 0.03) in students with parents with high education	Intervention had beneficial effect on BMI and BMIz in girls, but not boys; children of higher educated parents had more benefits from intervention—this needs attention in future to avoid further increase in social inequalities in overweight and obesity
Hølund et al. 1990/Denmark	Examine attitudes toward healthy foods; evaluate impact of health education program on attitudes	Cluster RCT crossover design × 4 months	127 14‐year‐old (8th grade) students from four schools (IG: 64, CG: 63)	14‐year‐old students learned about nutrition and dental health by teaching these topics to 10‐year‐old children	Theory of Cognitive Dissonance	Girls in both groups had more favorable pro‐health attitudes than boys; IG boys and girls had better attitudes toward food as means of health promotion than CG; no effect on taste of healthy foods	“Learning by teaching” succeeded in raising responsiveness to messages about healthy food as a means of health promotion; lack of impact on tastiness attitudes due to no taste experiences; in course; future programs should include taste experiences
Hoppu et al., 2010/Finland	To decrease the intake of sucrose, increase fiber intake, F/V consumption	Cluster randomized trial × one school year	469 8th grade students from 6 IG schools and 5 CG schools	Actions targeted at different actors, implemented at different times; actions focused on development of healthy food environment and nutrition education	Social Cognitive Theory	Frequency of consumption of rye bread increased (*p* = 03), sweets decreased (*p* = 0.006) among IG girls; sucrose fell among IG from 12.8% to 10.5% of the total energy intake (*p* = 0.01); fruit intake remained same in IG, decreased in CG (*p* = 0.04)	Sugar intake can be lowered by improving snack quality, but more difficult to increase fiber intake, F/V consumption unless school lunch content modified
Hrafnkelsson et al., 2014/Iceland	Assess effectiveness of a school‐based, multifocal intervention, on weight, fat percentage, cardiovascular risk factors, blood pressure	Cluster RCT × 2 years	283 2nd grade students from 6 schools (3 IG, 3 CG)	IG schools had integrated physical activity (60‐min per school day), nutrition education; parents, teachers, school food service staff all involved	N/R	No significant differences between IG and CG students in BP, percentage body fat, lipid profile, fasting insulin	This school‐based intervention with increased physical activity and healthy diet did not have a significant effect on common cardiovascular risk factors
Jensen et al., 2015/Denmark	Evaluate effect of multicomponent school‐based intervention, *Copenhagen School Child Intervention Study* on children's nutrient intake and whether intervention effect depended on maternal education level	3‐year pre–post study of two nonrandomized groups	307 children from 10 schools (IG: 184, mean age 6.8 years; CG: 123, mean age 9.5)	(1) Two additional PE sessions/week; (2) additional education of PE teachers; (3) improvement of schoolyard to facilitate physical activity; (4) parent involvement via newsletters; (5) establishment of school canteens selling healthy foods; (6) health education in curriculum	N/R	Changes in nutrient intake were observed in IG, mainly among children of mothers with a short education (< 10 years), including increase in dietary fiber (*p* = 0.01) protein intake (*p* = 0.05), decrease in fat (*p* = 0.09)	This school‐based intervention resulted in changes in dietary intake among children of mothers with a short education, a group that differs most from recommendations; results are particularly encouraging
Jørgensen et al., 2016/Denmark	Examine if parental involvement in a multicomponent nutrition program, *Boost*, was associated with adolescents' FV intake at follow‐up	National cluster RCT × 9 months	4 randomly selected schools from 10 communities, 1175 Danish 7th graders (13‐ year‐olds)	School: free FV in class/curricular activities; community: fact sheets for sports‐ and youth clubs; parents: 6 newsletters, 3 guided student‐parent curricular activities, events for student–parent	N/R	High, medium, and low parental involvement was found among 30.5%, 29.6%, and 39.4% of students, respectively, students with medium‐ and high level of parental involvement ate 47.5 and 95.2 g more F/V per day compared to students with low level/no parental involvement (*p* = 0.02)	Parental involvement in interventions may improve adolescents' F/V intake if implementation challenges can be overcome
Klepp and Wilhelmsen, 1993/Norway	Assess effectiveness of a 7th grade social norms/skills‐based educational program integrated into home economics courses	RCT × 4 months with f/up at 5 and 12 months	415 junior high school students	Education about increased F/V, whole‐wheat bread, low‐fat dairy, reduced high sugar/high fat snacks	Social learning model	IG females and males had significantly healthier eating behaviors than CG at 5‐month f/up; no difference at 12‐month f/up	Results demonstrate feasibility of integrating curriculum activities to modify student eating behavior in home economics courses
Klepp et al., 2005/Iceland, Denmark, Norway, Sweden plus four other non‐Nordic countries	Assess F/V use, determinants of use patterns among European schoolchildren and parents; develop/test strategies for promoting increased consumption of V/F in the *The Pro Children Project*	Group randomized trial with cross‐over design	National, representative samples of 11‐year‐old schoolchildren and parents (20 schools and minimum of 1300 children)	Key components: school education, food service provision, parent presentations, mass media, school health, sport clubs, grocery store program	ASE model (Attitudes, Social Influences Self‐Efficacy)	Girls eat F/V significantly more often than boys across all participating countries; no sex differences, re perceived F/V availability in and outside home setting; perceived availability associated with reported frequency of F/V intake	Experience suggests project will produce valid/reliable research instruments for assessing F/V consumption among children and parents; comparable programs can be implemented across geographic and cultural settings within Europe
Kristiansen et al., 2019/Norway	To improve vegetable intake in kindergarten‐based intervention, *BRA‐Study*	Block RCT × 6 months	633 kindergarten children (IG: 217; CG: 218)	Components focused on changing determinants: availability, accessibility, encouragement, role modeling in school/home settings	N/R	No significant overall effects were found for total daily vegetable intake or parental reported frequency/variety in vegetable intake	Lack of positive effects in home setting attributable to not knowing best strategies to involve parents; further research needed to understand best strategies to involve parents
Kristjansdottir et al., 2010/Iceland	To assess effect of school‐based intervention on diet	Cluster RCT	7–9‐year‐old in 2nd and 4th grade from 4 schools	Intervention aimed at determinants of food intake: knowledge, awareness, taste/preferences, self‐efficacy, parental influence; teacher intervention materials	Multiple school/home‐based intervention activities	F/V intake increased by 47% in the intervention schools (*p* < 0.001); most children in IG still did not meet guidelines on F/V at follow‐up	Intervention effective in increasing F/V intake by 47% from baseline, mirrored in nutrient intake
Laitinen et al., 2022/Finland	Investigate effects of a tailored teacher‐driven food education model, *Tasty School*, which provides tools for implementing food education on pupils' eating patterns and experiences	Quasi‐experimental design × 1 school year	1480 students from 5 IG and 10 CG schools	Using previous nutrition curriculum, education model to cover the entire primary school system (Grades 1–6, age 7–12 years) and was diversified with website, idea bank with learning materials, teacher training	Self‐Determination Theory	Students with balanced meals increased in IG schools where food education actively implemented (*p* = 0.027)	Healthy eating patterns can be promoted by active implementation of food education in primary schools
Laitinen et al., 2023/Finland	Assess teachers' experiences in Tasty School related to food education and school dining intervention	Quasi‐experimental design × 1 school year	130 class teachers (IG: 15, CG: 10)	Using previous nutrition curriculum, education model to cover the entire primary school system (Grades 1–6, age 7–12 years) and was diversified with website, idea bank with learning materials, teacher training	Self‐Determination Theory	Model highly acceptable, easily integrated into school environment; support from principals/colleagues most important facilitator of food education; lack of time a barrier	Commitment of whole school and principals' role is critical in implementation of food education; supporting factors must be strengthened, efforts made to reduce barriers
Larsen et al., 2021/Denmark	Investigate of a scaled‐up *FIFA‐11* “*health education through football*” on health knowledge and enjoyment	Scaled‐up cluster RCT × 11 week	3127 Danish children aged 10–12 years from 154 schools	2×/week 45‐min intervention includes health education, football drills, games with focus on hygiene, nutrition, physical activity, well‐being; health messages	N/R	IG/CG differences (*p* < 0.05) in overall health knowledge, health knowledge related to hygiene, nutrition, physical activity, well‐being; both girls/boys gave program moderate‐high scores for enjoyment	Health education through sport improved health knowledge related to hygiene, nutrition, physical activity, well‐being; similar improvements and enjoyment scores for both genders
Lundborg et al., 2022/Sweden	Assess if policy of nutritious school lunches free of charge for all pupils in Swedish primary schools between 1959–1969, *Swedish School Lunch Reform*, improve children's economic, educational, and health outcomes throughout life	Pre–post policy assessment of cohort	Historic data, births from 1973, weight, smoking behavior of women with data from Swedish Medical Birth Register	Policy imposed strict nutritional standards on meals served, which were to contain specified amounts of proteins, vitamins, calcium, iron, contain maximum fat content, provide a third of daily caloric need	N/R	School lunch program generated substantial long‐term benefits, where pupils exposed to program during their entire primary school period have 3% higher lifetime income; greater effect for pupils that were exposed at earlier ages and from poor households; also large, positive effects on height, early adult health, educational attainment	Study provides evidence that a universal program that provides school‐aged children with nutritious meals can be seen as an investment in their long‐run human capital, with high internal rates of return
Magnusson et al., 2012/Iceland	Assess effects of physical activity and dietary intervention program on body composition and cardiorespiratory fitness	Cluster RCT × 2 years	7‐year‐olds from 6 schools (IG: 151; CG: 170)	Teacher‐led daily implementation of intervention tactics, introduced, discussed during bimonthly meetings led by research team	N/R	None of the effect sizes of body composition were statistically significant	Results are inconclusive on fitness, with no significant effect on body composition
Marcus et al., 2009/Sweden	Assess efficacy of school‐based intervention, *STOPP*, to reduce overweight prevalence of overweight	Cluster RCT × 4 years	6–10‐year‐olds from 10 schools	Low‐fat dairy products, whole‐grain bread promoted, eliminate all sweets/sweetened drinks; Physical activity (PA) increased by 30 min day during school time, sedentary behavior restricted in after school care	N/R	Prevalence of overweight and obesity decreased in IS compared with increase in CS (*p* < 0.05); larger proportion of children initially overweight reached normal weight in IG compared to CG (*p* = 0.017)	School‐based intervention can reduce overweight/obesity in 6–10‐year‐old children, may affect eating habits at home; intervention effect possibly due to healthy eating habits at school and home rather than increased *p* levels
Nyberg et al., 2016/Sweden	Evaluate effective of parent support program, *Healthy School Start Study II*, to promote healthy dietary and physical activity habits, and prevent overweight/obesity	Cluster RCT × 6 months with wait‐list control group	13 schools, 31 classes (IG: 16; CG: 15)	Health information, two sessions of Motivational Interviewing with parents; teacher‐led classroom activities	Social Cognitive Theory	Significant intervention effects for consumption of unhealthy foods (*p* = 0.01), unhealthy drinks (*p* = 0.01); at f/up effect on intake of unhealthy foods was sustained for boys (*p* = 0.03); no effect on physical activity	Positive effects short‐lived; program probably needs to be prolonged and/or intensified in order to obtain stronger and more sustainable effects
Outzen et al., 2023/Denmark	Evaluate effect of *FOODcamp* educational intervention on dietary habits	Cluster RCT × 1 school year	11–13‐year‐old from 9 schools; IG: 16 classes (322 children); CG: 16 classes (267 children)	5‐day educational program to promote healthy dietary habits; activities designed to develop cooking skills, improve understanding of interconnection between food, health, well‐being, and nature	N/R	No statistically significant effects were found on take of food groups eaten regularly (vegetables, fruit, vegetables/fruit/juice combined, or meat) (*p* > 0.05)	Study found no effect of the educational intervention FOODcamp on dietary intake; cannot exclude that dietary behavior affected later in life when children responsible for their own cooking
Øverby et al., 2012/Norway	Analyze changes in consumption of unhealthy snacks and assess if school fruit program, *Fruits and Vegetables Make the Marks*, reduces frequency of unhealthy snacks	Nonrandomized comparison study × 7 years	All 6th–7th grade students in 48 schools participating in national free fruit program	Students receive a piece of fruit or a carrot each school day	N/R	Frequency of unhealthy snacks decreased from 6.9 to 4.6 times/week (*p* < 0.001) decrease largest in schools in free school fruit program (22.8 times/week); significant in reducing frequency of unhealthy snack in children of parents without higher education (*p* = 0.004)	Results show that children attending school with fruit program have decreased frequency of unhealthy snack consumption, indicating increased fruit intake does replace unhealthy snacks
Prell et al., 2005/Sweden	Examine effect of two school‐based interventions on adolescents' fish consumption/knowledge	Controlled pre–post design × 1 school year	8th graders from 3 schools, *N* = 228 (CG: 83; IG (school lunch), 58; SL + HE 87)	School lunch intervention made changes in school canteen; school lunch + home economics intervention (SL + HE) consisted of changes in syllabus	N/R	Consumption increased significantly in SL + HE group; significant positive knowledge changes in both intervention groups, but not in controls	Results suggest that dietary change achieved by modifying conditions in school canteen together with changing home economics syllabus; shows importance of school in promotion of dietary change among adolescents
Puska et al., 1982/Finland	Assess whether health behavior and risk factors can be influenced by described intervention program, *North Karelia Youth Project;* assess whether an intervention in all county schools in the county would have similar effects	Three level pre–post program assessment	851 7th grade students in 6 randomly chosen schools × 2 years compared to CG school	Two intervention levels: (a) intensive direct intervention (II) in two schools, and (b) a general county‐wide intervention (CI)	N/R	Significant difference in cholesterol decline between both II (*p* < 0.01), CI (*p* < 0.05), and CG; mean amount of fat from milk and butter changed among and among girls (both *p* < 0.01 between II and R); intervention had no effect on blood pressure; smoking increase was more than double in the CG schools compared with the II schools for both boys (*p* < 0.001) and girls (*p* < 0.05)	Program implementation good, well‐received by children, teachers, and parents; relatively inexpensive, could be easily carried out elsewhere in the country; program effective in preventing smoking increase; nutritional program was more effective among girls than boys; effects obtained by broad‐ranged intervention teaching practical skills/influencing social and physical environment, not emphasizing health knowledge
Räihä et al., 2012/Finland	Evaluate effects of nutrition health intervention, *From Puijo to the World with Health Lunch*, on pupils' nutrition knowledge and eating	Pre–post × 2 years	13–14‐year‐olds from 4 schools; IG: 106; CG: 88	Intervention involved curriculum (biology, health, home economics) school nurses, counseling, parents	N/R	Nutrition knowledge, and use of healthy foods increased significantly in IG compared to CG (*p* = 0.017; *p* = 0.008)	Interventions required continuous cooperation of health‐promoting experts, school staff, families, pupils; need future long‐term evaluations on pupils' long‐term knowledge/eating habits
Randby et al., 2024/Norway	Examine effectiveness of a multistrategy implementation intervention to increase adherence to Norwegian national school meal guideline	Type II hybrid effectiveness trial, with pre–post nonequivalent control groups	Students aged 6–13 years (Grades 1–7) from 14 schools (IG: 9; CG: 14)	Tested three arms: internal facilitators as agents of practice change (teachers appointed as ambassadors); educational meeting for all participants (principals, food ambassadors, and after‐school leaders); training of food ambassadors/after‐school leaders	N/R	Significant difference of four percentage points in change scores between IG and CGs (*p* = 0.003).	Low intensity school‐based intervention using trained teachers as facilitators can increase adherence to national school meal guideline among Norwegian primary schools
Ren et al., 2021/Denmark	Evaluate changes in intake of selected foods/beverages during multicomponent school‐ based physical activity intervention, *Copenhagen School Child Intervention Study*; investigate if changes modified by SES	Two‐group comparison pre–post × 3 years	307 children (intervention group: 184; comparison group: 123)	6‐part intervention: (1) two additional 45 min/week PE lessons; (2) education of PE teachers; (3) improvement in schoolyard environment, (4) introduction of healthy school canteens, (5) parental involvement; (6) health education in curriculum.	N/R	IG increased intake of whole‐grain bread compared to CG (*p* = 0.04). CG children from low SES families decreased fruit intake compared to IG (*p* = 0.006).	Study found no convincing effect of introducing a multicomponent intervention on dietary intake except a small beneficial effect on whole‐grain bread consumption; beneficial effects in fruit intake found particularly among children from low SES families
Rohde et al., 2017/Denmark	Evaluate impact of a 15‐month intervention, *Healthy Start*, on dietary intake conducted among obesity‐prone normal‐weight preschool children	RCT × 15 months	285 preschool children with birth weight ≥ 4000 g, or high maternal prepregnancy BMI, or low maternal education level (< 10 years of school)	IG assigned a health/nutrition consultant, with up to 10 meetings who counseled on 8 Dietary Guidelines	N/R	IG had lower energy intake after the 15‐month intervention (*p* = 0.02) compared to CG; lower intakes of carbohydrates and added sugar (*p* = 0.002, *p* = 0.01)	Intervention resulted in lower energy intake, particularly from carbohydrates and added sugar suggesting dietary intake can be changed in a healthier direction in children predisposed to obesity
Ryom et al., 2022/Denmark	Investigate effects of health promotion intervention *11 for Health in Denmark*	RCT × 11 weeks	1122 5th grade/10–12‐year‐old ethnic minority schoolchildren (IG: 944; CG: 178)	Two 45‐min/week football drills, small‐sided games, health education	N/R	Compared to CG, IG had more positive effect on health knowledge related to hygiene (*p* < 0.05) nutrition (*p* < 0.001), physical activity, overall health knowledge (*p* < 0.05), similar effect for girls and boys	Program had positive effect on health knowledge of ethnic minority background schoolchildren
Sørensen et al., 2015/Denmark	Investigate if serving healthy school meals using *New Nordic Diet* influenced concentration and school performance	Clustered RCT crossover trial × 6 months	739 8–11‐year‐olds from 46 classes in 9 schools	Compared healthy school meal program consisting of lunch meal, a midmorning and afternoon snack served on each school day free of charge with usual packed lunch from home	N/R	Intervention did not influence concentration performance or processing speed; decrease in error percentage was smaller (*p* < 0.001) during intervention than control; percentage of correct sentences improved (*p* < 0.001); no effect on overall math performance	School meals did not affect concentration performance, but improved reading performance, a complex cognitive activity; findings are worth examining in future trials
Sørensen et al., 2016/Denmark	Explore if cognitive effects of school meals program of New Nordic Diet differed by gender, household education reading proficiency	Clustered RCT crossover trial × 6 months	739 8–11‐year‐olds from 46 classes in 9 schools	Compared healthy school meal program consisting of lunch meal, a midmorning and afternoon snack served on each school day free of charge with usual packed lunch from home	N/R	Intervention effect on test outcomes stronger in boys, in children from households with academic education and in children with normal/good baseline reading proficiency; resulting in increased socioeconomic inequality in reading performance and reduced inequality in impulsivity; gender difference decreased in reading and increased in impulsivity; gap between poor and normal/good readers increased	Effects of healthy school meals on reading, impulsivity and inattention modified by gender, household education, and baseline reading proficiency; differential effects might be related to environmental aspects of intervention and deserve investigation in future trials
TeVelde, Wind et al., 2008/Norway among other non‐Nordic countries	Evaluated effects of multicomponent intervention, *Pro Children Study*, on mothers' intake levels	Group RCT × 1 school year	Mothers of 11‐year‐olds	Combined FV curriculum with efforts to improve FV availability at schools and home	N/R	No intervention effect on mothers' F/V intake after 1‐year and 2‐year follow‐up	Intervention did not increase mothers of F/V consumption which might be explained by low involvement in project
TeVelde, Brug, et al., 2008/Norway among other non‐Nordic countries	Evaluate effects of *Pro Children* intervention on children's F/V intake after 1 and 2 years of follow‐up	Group RCT × 1 school year	1472 10–11‐year‐old students from 62 schools	Combined FV curriculum with efforts to improve FV availability at schools and home	N/R	Significant intervention effects for FV intake found at first follow‐up in total sample; at 1‐year significant impact only observed in Norway; positive effects on FV intake occurred both at and outside school	Intervention is promising to promote F/V intake in European schoolchildren, but mainly fruit; strategies need to be developed that can improve implementation, have an impact on vegetable intake secure sustained effects
Vik et al., 2020/Norway	Investigate if *School Meal Project*, providing a free healthy school meal, resulted in higher F/V intake, lower intake of unhealthy snacks	Nonrandomized trial × 1 school year	164 schoolchildren, 10–12‐year‐olds (IG: 55 CG: 109)	Free school meal × 1 year prepared in accordance with current Norwegian dietary guidelines	N/R	Free healthy school meal associated with higher weekly F/V intake in IG compared to CG; no other intervention effects found; free meal not associated with lower weekly intake of unhealthy snacks	Given inadequate vegetable intake among children, even moderate improvements have public health relevance; free healthy school meals for all schoolchildren could be beneficial
Waling et al., 2010/Sweden	Evaluate the impact of a food and physical activity intervention on energy and macronutrient intake in overweight and obese children	Randomized open trial × 1 year	92 overweight or obese 8–12‐year‐old children	14 group sessions with different themes of food and physical activity	N/R	Compared to CG, IG had 11% decrease in energy intake relative to total energy expenditure (*p* = 0.020), decreased daily cholesterol intake (*p* = 0.006), lower increase in fat intake (*p* = 0.035); in contrast carbohydrate intake decreased in CG, did not change in IG (*p* = 0.010); IG increased sucrose intake (*p* = 0.036)	Despite comprehensive intervention, only modest effects achieved with respect to reduced energy intake and improved macronutrient intake
Wind et al., 2008/Norway among other non‐Nordic countries	Investigate degree of F/V *Pro‐Child Study* implementation, analyze how these factors associated with changes in F/V intake	Cluster RCT of schools × 1 year	10–13‐year‐olds from 117 classes in 62 schools over 3 countries	Curriculum was 16 lessons guided by worksheets and web‐based computer feedback tool that children completed	N/R	Teacher‐reported level of curriculum implementation, students' appreciation of project were important determinants	Results point to importance of optimal implementation of a school curriculum

Abbreviations: BMI, body mass index; BMIz, BMI‐for‐age z‐score; CG, control group; IG, intervention group; FH, Food and Health; F/V, fruits and vegetables; LF, laser fluorescence; NND, New Nordic Diet; NR, not reported; PA, physical activity; PE, physical education; SES, socioeconomic status; WTHR: waist‐to‐height ratio.

### 3.1 Goals and Study Design Characteristics of Included Interventions

Although the 53 included studies varied in design and setting, they shared common objectives that can be grouped into these overarching categories: (a) increasing fruit and vegetable (F/V) consumption [16, 23, 24, 25, 26, 27, 28, 29, 30, 31, 32, 33, 34], (b) improving general dietary and healthy behaviors and food literacy [13, 19, 22, 35, 36, 37, 38, 39, 40, 41, 42, 43], (c) reducing sugar and discretionary food intake [44, 45], (d) addressing weight status and obesity prevention [15, 20, 46, 47, 48, 49, 50], (e) enhancing nutrition knowledge and attitudes [17, 18, 21, 51, 52], and (f) improving school meal quality and sustainability [53, 54, 55, 56, 57, 58, 59, 60, 61].

Over 30 studies employed cluster‐randomized controlled trials (cluster‐RCTs) [13, 15, 18, 21, 23, 24, 25, 26, 27, 28, 29, 30, 31, 34, 37, 38, 39, 41, 43, 44, 45, 47, 48, 49, 50, 52, 53, 54, 56, 57, 62, 63]. Approximately ten studies used quasi‐experimental or controlled pre–post designs [14, 16, 17, 22, 35, 40, 42, 55, 59, 61, 64]. For example, the Stockholm County Implementation Programme was a 2‐year quasi‐experimental intervention [14], while the Tasty School intervention in Finland applied a similar design [35]. Long‐term cohort or policy evaluations were also conducted [16, 23, 60]. Several studies employed mixed‐methods designs to assess both quantitative outcomes and implementation processes [19, 46, 65].

### 3.2 Sample Sizes and Participant Characteristics

Sample sizes across the 53 reviewed studies varied considerably, ranging from small pilot interventions with < 100 participants [52, 55] to large national or international cohorts involving thousands of students [46]. A total of 26 studies included primary school students (6–11/12 years) in their intervention samples [13, 14, 15, 20, 21, 22, 23, 27, 29, 32, 33, 38, 39, 40, 41, 43, 44, 46, 48, 49, 51, 53, 57, 61, 63, 65]. Other studies focused on adolescents in lower secondary school [13, 16, 17, 18, 23, 24, 25, 28, 31, 35, 38, 39, 41, 42, 45, 47, 54, 55, 58, 62, 64]. Multinational and large‐scale national studies were common; for example, the IDEFICS study included over 16,000 children aged 2–9.9 years from 8 European countries [46], while the Pro Children project reached thousands of 10–13‐year‐old students and their parents across Europe [27, 28, 29, 30]. Some interventions extended participation beyond students to include teachers, school staff, or parents [19, 30, 36, 39, 65], recognizing the broader influence of adults in shaping the school food environment.

### 3.3 Intervention Characteristics in Included Studies

The included school‐based interventions utilized a wide variety of strategies to promote healthy eating behaviors among children and adolescents. Most were multicomponent in nature, combining individual student‐focused education, changes to the school environment, and engagement with families or the wider community. Many of these interventions (*n* = 12) focused on improving the school food environment through the provision of free or healthy school meals and snacks [16, 23, 24, 25, 26, 32, 33, 53, 55, 56, 57, 59, 60, 64]. Other intervention included nutrition education or integrated it into the school curriculum (*n* = 13) [18, 27, 28, 29, 30, 35, 37, 41, 42, 52, 54, 55, 62]. Active involvement of parents and community members was part of 12 intervention studies [13, 17, 22, 30, 31, 38, 39, 40, 44, 46, 49, 65]. There were also several programs combining physical activity with broader lifestyle changes to ensure a comprehensive intervention [13, 20, 21, 40, 49, 51, 63, 65]. Some studies included policy‐ or environmental‐level approaches or multicomponent intervention aimed at creating supportive environments for healthy behaviors [13, 15, 17, 19, 34, 38, 41, 42, 59, 60, 61].

### 3.4 Theoretical Frameworks

Of the 53 included studies, just over one‐third (*n* = 18) explicitly reported using a theoretical framework to guide intervention design, implementation, or evaluation [13, 14, 24, 27, 34, 35, 36, 38, 39, 44, 45, 48, 52, 54, 55, 58, 59, 62]. Among these, Social Cognitive Theory was the most frequently applied [24, 44, 45, 47, 59]. The Socio‐Ecological Model of Health was employed by Elinder et al. [14], while Hølund [52] based their work on the Theory of Cognitive Dissonance. Social Learning Theory guided the intervention by Klepp and Wilhelmsen [62], and the ASE model (Attitude, Social Influence, and Self‐Efficacy) was used by Klepp et al. [27] in a multicountry study. More recently, the Self‐Determination Theory informed interventions by Laitinen et al. [35, 36]. Hence, the majority of studies (approximately two‐thirds) did not report any theoretical foundation.

### 3.5 Outcomes

The reviewed interventions consistently demonstrated positive impacts on dietary behaviors, nutrition knowledge, and in some cases, physical health indicators, though effect sizes and sustainability varied. Many programs succeeded in increasing fruit and vegetable (F/V) intake [16, 22, 25, 26, 27, 29, 32, 33, 41, 62], though some found limited or no sustained change [24, 34, 37]. Improved nutrition knowledge or attitude was widely reported [17, 18, 19, 21, 51, 52, 63, 64], often translating into healthier food choices. For example, programs combining education and environmental change, like school fruit schemes or free meal initiatives, were associated with higher F/V intake and reduced unhealthy snack consumption [16, 26, 44]. Positive changes in broader dietary patterns included reduced discretionary food and sugar intake [35, 39, 40, 44, 45, 47, 50, 53, 54, 55], increased whole grain consumption [22], and improved breakfast quality [56]. Some studies highlighted equity impacts; children from lower‐SES backgrounds or with less‐educated parents sometimes benefited more [16, 40]. However, other interventions inadvertently widened inequalities, such as in academic performance linked to school meals [58].

In terms of physical health outcomes, reductions in BMI or obesity prevalence and/or improvement in biochemical markers were seen in several studies [38, 43, 48], though others found no significant changes in these parameters [15, 23, 46, 49]. Positive effects on physical fitness or body composition were more likely when physical activity was integrated [20, 39, 40]. Implementation fidelity and school engagement emerged as critical success factors. Achievement of sustained behavior change depended on the support provided from staff and families [13, 28, 31, 36, 61, 65]. Conversely, interventions struggled where time, resources, or leadership were lacking [14, 36, 65]. A few studies showed long‐term impacts beyond school years, with evidence linking healthier school meals to better adult health, educational attainment, and income [60]. However, one study reported no long‐term effect among parents [30].

## 4 Discussion

The discussion across the 53 articles reviewed for Nordic school‐based interventions highlights several important themes relevant to nutrition promotion and public health outcomes. Some studies reported positive dietary changes [50, 54], particularly increased fruit and vegetable (F/V) consumption [29, 33, 45], more when programs included free school meals or fruit schemes [16, 23, 25, 26, 32]. These low‐cost, school‐based strategies were especially effective among children from lower socioeconomic backgrounds [22, 40], though disparities persisted or even widened in some cases [26, 58]. For instance, Grydeland et al. [38] emphasized the risk of increasing social inequality in overweight prevention unless interventions are designed with equity in mind. Equity impact is strongest in Finland and Sweden, but not as strong in Denmark and Norway.

The combination of strong and enduring national nutrition recommendations via the NNR, with universal free and highly subsidized access to fruits and vegetables in the school and welfare programs and the additional environmental messaging makes the Nordic countries stand out in fruits and vegetable promotion, compared with OECD countries. For example, the EU has a School Fruit and Vegetable Scheme that is used in many industrialized countries, but Nordic countries go beyond the EU minimum in scale and integration. Other OECD countries tend to rely on time‐limited projects, parental payment, or voluntary participation, which tends to produce smaller and less equitable effects on establishing healthy food consumption [66]. These Nordic countries have the similar messaging of “5‐a‐Day” fruit and vegetable consumption as the major OECD countries, with Norway adding berries [67]. Given the success that the Nordic countries are having, they realize more progress needs to be made in sustaining vegetable intake gains, translating school‐time intake into total daily consumption, and maintaining political support for universal schemes.

Multicomponent and/or theory‐driven interventions were particularly effective when combining nutrition education with environmental or behavioral strategies [13, 35, 39, 44, 63]. Integrating physical activity, like football‐based or active learning approaches, further enhanced knowledge and health outcomes [20, 21, 51]. Other interventions, such as school breakfast or lunch programs, contributed to improved dietary quality [55] and, in some cases, academic performance [53, 56, 57]. Since the 1980s, the Nordic countries have continued to evolve their approaches in school nutrition education such as moving from micronutrient sufficiency toward whole‐diet quality and food culture and integrating meals and education by making food a part of learning and not just feeding. Furthermore, nutrition education in these countries has responded to the teaching across the curriculum movement by embedding food and nutrition messages naturally into the home economics and health education subjects [68].

Importantly, the study by Lundborg et al. [60] provides evidence that universal program that provides school‐aged children with nutritious meals could be perceived as an investment in their long‐run human capital, with high internal rates of return. Integrating curriculum activities to modify students dietary behavior have been shown to be feasible and recommended [28, 62]. However, not all programs were successful. Some reported limited or no significant impact on students' dietary habits [24], BMI [30, 43, 46], or health markers [15, 37, 49, 59]. These results often stemmed from weak implementation fidelity, insufficient duration, or lack of stakeholder engagement [14, 34, 65]. Furthermore, several studies stressed the importance of contextual tailoring, particularly involving parents [27, 29, 48, 64], school staff [29, 36], and community actors, as well as healthy school canteen [64] and providing meal at schools [45] to sustain engagement and impact [17, 31]. Sweden and Finland are two countries of note that show that the universality of health nutrition programming in schools is bolstered by a municipal commitment in implementation with national guidelines [69, 70].

However, a few interventions offered novel strategies. Abdollahi et al. [18] tested an educational escape game to promote plant‐based knowledge, and Beinert et al. [19] included learning specific tasks related to food and nutrition; while Hølund [52] used a peer‐led model, with older students teaching younger children about nutrition. These creative approaches may help improve motivation and learning outcomes when implemented at scale. Meanwhile, school‐wide initiatives that reshaped menus or incorporated climate goals reflected growing attention to sustainable food systems, although impact evidence remained modest [59]. This work is particularly salient as the latest NNR published in 2023 focused on integrating environmental aspects [9]. Finally, Puska et al. [42] concluded that the intervention was well‐received by children, teachers and parents, qualifying it as effective and easy to be carried out elsewhere in Finland.

Surprisingly, mass media and social media are absent from these interventions. While the school environment is considered a closed system in many ways, and the social interaction of the students with their families, friends, and neighbors is different than the interactions is school, much could be gained by implementing interventions that have some social media or mass media component. One mass intervention by Klepp et al. [27] did expand their intervention components to school education, food service provision, parent presentations, mass media, school health, sport clubs, and grocery store programs. Given the high levels of digital access, media literacy, and public trust in health institutions across Nordic countries, the limited application of social marketing approaches in school nutrition programs warrants further use and investigation and targeted development in future intervention research.

This review illustrates some general areas of focus among the five nations such as universal provisions, curriculum innovation, intervention research, and equity awareness. Finland and Sweden are front runners for all four focus areas mentioned above. Denmark not only has the largest number of studies published among the five nations but set up several rigorous experimental designs such as the RCT OPUS School Meal Study [53].

This review also illustrates that these countries have a robust set of signature school food and health curricula, short term programs, campaigns, and guidelines at their disposal and continue to evaluate them. Of the 53 studies, 36 involved one of these signature programs. Ones most often evaluated are *The New Nordic Diet* (Denmark), *HEalth In Adolescents* (Norway), *Fruits and Vegetables Make the Marks* (Norway), and *FIFA‐11 health education through football* (Denmark). Signature interventions were most popular in Norway and Denmark, with three studies involved in Finland and only one of these signature programs used in an Iceland study.

## 5 Conclusion

While Nordic countries have a relatively high socioeconomic status population, they are challenged by the rising prevalence of obesity and overweight among young people. This scoping review (1980 to May 2025) shows that there are healthy efforts to effectively increase nutrition education programs in schools, but some gaps in activity exist, given this almost four‐decade review. Some schools have the advantage of a national nutrition education framework that gives consistency to the nature and extent of nutrition promotion, but all schools have found ways to include nutrition education programs in the curriculum.

Most programs were multicomponent in nature, combining individual student‐focused education, changes to the school environment, and engagement with families or the wider community. Of the 53 included studies, just over one‐third (*n* = 18) explicitly reported using a theoretical framework to guide intervention design, implementation, or evaluation. The reviewed interventions consistently demonstrated positive impacts on dietary behaviors, nutrition knowledge, and in some cases, physical health indicators, though effect sizes and sustainability varied.

On the one hand, using school sites for nutrition education appears both economical and feasible, given that students are a captive audience and there are some efficiencies in implementation, especially in gaining knowledge acquisition. However, this review identified barriers that challenge school‐based nutrition education programs such as fidelity to evidence‐based programs, shifting school schedules affecting instruction, and mixed stakeholder commitment from administrators, teachers, and parents. Government or private entity provisions of meals or snacks have a significant effect on improvement of youth nutrition, especially for students with limited familial resources.

This review helps validate the historic account of the NNR report of 2023, now in its 6th edition (1980, 1989, 1996, 2004, 2012, and 2023), that there has been a gradual expansion of the focus on school‐based nutrition education from nutrient adequacy to health promotion, to sustainability. Each is an add on, not a replacement and the latter's sustainability relates to a range of environmental issues. Furthermore, the interventions have moved from micronutrient sufficiency toward whole‐diet quality and food culture. The region's practice shows that nutrition education is most effective when combined with guaranteed access to healthy meals, supported by sustained governance, and curricular integration.

Thus, the following recommendations emerge from the review:

- Providing free or universally subsidized school meals reduces socioeconomic disparities in diet quality, participation, and social inclusion, particularly in Finland and Sweden where meals are statutory rights. Free meals function as a social equalizer and support consistent meal consumption across income groups. Interestingly, across Nordic school meal research, stigma reduction is rarely framed as a primary justification for school nutrition programs. This contrasts with much of the OECD literature, where means‐tested meals and food insecurity make stigma a moderate to central concern.
- Naturally, schools should consistently adhere closely to and align menus with the NNR, emphasizing whole grains, vegetables, fruits, fish, and limited saturated fat and added sugar which should give a higher availability of nutrient‐dense foods.
- Messaging should focus beyond nutrient value to boost student acceptance of healthy meals by being attentive to taste, respectful of student autonomy of choice, and an agreeable social environment, for example, student interaction and belonging.
- Interventions optimizing menus for lower climate impact show some promise, but the concept and implementation are evolving as the science, and sociopolitical influences change or are updated.
- Interventions involving school leadership, catering staff, teachers, parents, and community, while adding to the complexity and cost of programming, should show better outcomes and sustainability.

## Author Contributions

**Basil H. Aboul‐Enein:** conceptualization, writing – original draft, methodology, validation, visualization, writing – review and editing, software, project administration, supervision, resources. **Stephen Gambescia:** writing – original draft, investigation, validation, visualization, writing – review and editing, data curation. **Nada Benajiba:** writing – original draft, writing – review and editing, visualization, validation, formal analysis, data curation. **Norah Eid Aljohani:** writing – original draft, writing – review and editing, visualization, validation, formal analysis, data curation. **Teresa Keller:** writing – original draft, writing – review and editing. **Patricia J. Kelly:** investigation, writing – original draft, methodology, validation, visualization, writing – review and editing, formal analysis, project administration, data curation, supervision, resources.

## Funding

The authors have nothing to report.

## Ethics Statement

The authors have nothing to report.

## Conflicts of Interest

The authors declare no conflicts of interest.

## 1 Transparency Statement

The lead author Basil H. Aboul‐Enein affirms that this manuscript is an honest, accurate, and transparent account of the study being reported; that no important aspects of the study have been omitted; and that any discrepancies from the study as planned (and, if relevant, registered) have been explained.

## Supporting information

Supplementary Material.

## Data Availability

All data generated and analyzed during this review are included in the published review article.
