# Supplementary material for: A Regional Scoping Review of School‐Based Nutrition Interventions Conducted Across Nordic Countries
Source: Health Sci Rep. 2026 Apr 5;9(4):e72279. doi: 10.1002/hsr2.72279 (PMC13052336; doi:10.1002/hsr2.72279)
Supplement: Supplementary file 1 — Supplementary Material. [file HSR2-9-e72279-s001.docx]

**Supplementary material**

***Scopus Database***

“School” [All Fields]; “school-based” [All Fields]; “elementary” [All Fields]; Primary” [All Fields]; “Secondary” [All Fields]

AND

“nutrition” [All Fields]; “diet” [All Fields]

AND

“Intervention” [All Fields]; “Program” [All Fields]; “Education” [All Fields]

AND

“Norway”; “Finland”; “Iceland”; “Denmark”; “Sweden” [All Fields]
